# *In Silico* Identification of Potent PPAR-**γ** Agonists from Traditional Chinese Medicine: A Bioactivity Prediction, Virtual Screening, and Molecular Dynamics Study

**DOI:** 10.1155/2014/192452

**Published:** 2014-05-26

**Authors:** Kuan-Chung Chen, Calvin Yu-Chian Chen

**Affiliations:** ^1^School of Pharmacy, China Medical University, Taichung 40402, Taiwan; ^2^School of Medicine, College of Medicine, China Medical University, Taichung 40402, Taiwan; ^3^Department of Biomedical Informatics, Asia University, Taichung 41354, Taiwan

## Abstract

The peroxisome proliferator-activated receptors (PPARs) related to regulation of lipid metabolism, inflammation, cell proliferation, differentiation, and glucose homeostasis by controlling the related ligand-dependent transcription of networks of genes. They are used to be served as therapeutic targets against metabolic disorder, such as obesity, dyslipidemia, and diabetes; especially, PPAR-*γ* is the most extensively investigated isoform for the treatment of dyslipidemic type 2 diabetes. In this study, we filter compounds of traditional Chinese medicine (TCM) using bioactivities predicted by three distinct prediction models before the virtual screening. For the top candidates, the molecular dynamics (MD) simulations were also utilized to investigate the stability of interactions between ligand and PPAR-*γ* protein. The top two TCM candidates, 5-hydroxy-L-tryptophan and abrine, have an indole ring and carboxyl group to form the H-bonds with the key residues of PPAR-*γ* protein, such as residues Ser289 and Lys367. The secondary amine group of abrine also stabilized an H-bond with residue Ser289. From the figures of root mean square fluctuations (RMSFs), the key residues were stabilized in protein complexes with 5-Hydroxy-L-tryptophan and abrine as control. Hence, we propose 5-hydroxy-L-tryptophan and abrine as potential lead compounds for further study in drug development process with the PPAR-*γ* protein.

## 1. Introduction

The peroxisome proliferator-activated receptors (PPARs) belonged to the nuclear receptor superfamily of ligand-inducible transcription factors. They are “fatty acid sensors” related to regulation of lipid metabolism, inflammation, cell proliferation, differentiation, and glucose homeostasis by controlling the related ligand-dependent transcription of networks of genes [1–3]. There are three different isoforms of PPARs in mammal, which are PPAR-*α*, PPAR-*γ*, and PPAR-*δ*/*β*. They have different tissue distributions and responses to different ligands [4–6]. PPARs are used to be served as therapeutic targets against metabolic disorder, such as obesity, dyslipidemia, and diabetes; especially, PPAR-*γ* is the most extensively investigated isoform for the treatment of dyslipidemic type 2 diabetes [7–10]. It is a well-known receptor located in fat for antidiabetic insulin sensitizers and has the functions related to adipogenesis, lipogenesis, and glucose homeostasis [11–13]. In rat stroke models, PPAR-*γ* has been served as a brain protector against ischemic cerebral infraction [14].

Nowadays, increasing numbers of drug are designed with a target protein against a specific disease [15–18], as increasing numbers of distinct mechanism of diseases have been identified by the researches [19–26]. Recently, the compounds from traditional Chinese herb have been proven to have the therapeutic effects [27–30]. In previous researches, many compounds of traditional Chinese medicine (TCM) have been indicated as potential candidates of lead compounds against cancer [31–34], neuropathic pain [35], stroke [36, 37], and virus infection [38, 39].

In the former study, we aim to detect potential candidates from TCM compounds as agonists targeting PPAR-*α*, PPAR-*δ*, and PPAR-*γ* [40]. However, a compound which had a higher binding affinity with target protein may not always obtain a higher bioactivity. In this paper, we aimed to focus on the target protein of PPAR-*γ* and filter TCM compounds using bioactivities predicted by three distinct prediction models before the virtual screening. The molecular dynamics (MD) simulations were also utilized to investigate the stability of interactions between ligand and PPAR-*γ* protein in the docking pose under dynamic conditions. We attempt to identify the potent TCM compounds with higher bioactivities and binding affinity for PPAR-*γ* protein and discuss the functional group of these candidates and common binding residues of PPAR-*γ* protein in their docking pose.

## 2. Materials and Methods

### 2.1. Data Collection

After TCM compounds from TCM database, Taiwan [41], were filtered by Lipinski et al.'s rule of five [42], a total of 9,029 nonduplicate compounds were prepared by Prepare Ligand module in Discovery Studio 2.5 (DS2.5) to adjust the ionization state to physiological setting for virtual screening. For calculating the pharmacokinetics properties, ADMET Descriptors model in DS2.5 was employed to calculate the aqueous solubility, CYP2D6 binding, hepatotoxicity, and plasma protein binding (PPB) as absorption, distribution, metabolism, excretion, and toxicity (ADMET) properties for each compound.

The X-ray crystallography structure of the human peroxisome proliferator-activated receptor gamma (PPAR-*γ*) protein was obtained from RCSB Protein Data Bank with PDB ID: 3K8S [43]. After protein preparation, the chain A of PPAR-*γ* protein was used as target protein for virtual screening, and T2384, cocrystallized in PPAR-*γ* protein, was used as control.

### 2.2. Biological Activity Prediction Using Multiple Linear Regression (MLR), Support Vector Machine (SVM), and Bayes Network Toolbox (BNT) Models

For the prediction of biological activity for the TCM compounds, three distinct prediction models were constructed with the pEC_50_ (log(1/EC_50_)) value of 20 compounds from Rikimaru et al.'s study [2] as training set. The genetic function approximation module [44] of DS 2.5 was utilized to determine the suitable molecular descriptors for constructing the prediction models, and the fitness of individual model was estimated by square correlation coefficient (*R* ^2^). Cross-validation test was used to validate the prediction model. For three distinct prediction models, multiple linear regression and Bayes network toolbox were performed using MATLAB, and support vector machine was performed using LibSVM developed by Chang and Lin [45].

### 2.3. Docking Simulation

For virtual screening, LigandFit protocol [46] in DS 2.5 was employed to dock each compound into an active site using a shape filter and Monte Carlo ligand conformation generation, and each docked pose was minimized with Chemistry at HARvard Macromolecular Mechanics (CHARMM) force field [47] and evaluated with a set of scoring functions. In addition, LigPlot v.2.2.25 program [48] was employed to identify the interactions between protein and ligand in each docking pose.

### 2.4. Molecular Dynamics Simulation

Before the molecular dynamics simulation by Gromacs [49], each protein-ligand complex in docking pose has been reprepared. Each ligand was reprepared by SwissParam program [50], and the protein was reprepared with charmm27 force field by Gromacs. The protein-ligand complex was solvated using a water model of TIP3P with a minimum distance of 1.2 Å from the complex and then minimized by steepest descent algorithm [51] with maximum of 5,000 steps. Then a single 10 ps constant temperature (NVT ensemble) equilibration was performed using Berendsen weak thermal coupling method followed by a 40 ns production simulation. For each MD simulation, it adopts the particle mesh Ewald (PME) option with a time step of 2 fs. A series of protocols in Gromacs were employed to analyze the MD trajectories.

## 3. Results and Discussion

### 3.1. Biological Activity Predictions

The genetic approximation algorithm determined the six optimum molecular descriptors for constructing prediction models with 20 compounds of training set. The selected descriptors were ES_Sum_sssCH, ES_Count_aaN, BIC, IAC_Mean, CHI_3_P, and JY. These six optimum molecular descriptors can be broadly divided into two groups, which are electronic and special topological descriptors. For electronic topological descriptors, it includes ES_Sum_sssCH, ES_Count_aaN for calculating the sums of the electrotopological state (E-state) values and the counts of each atom type, respectively. For special topological descriptors, BIC and IAC_Mean are bonding information content and mean information of atomic composition, which both belong to Graph-Theoretical InfoContent descriptors [52]. CHI_3_P is a Kier and Hall molecular connectivity index [53]. JY is a Balaban index [54]. According to these selected descriptors, the functional formula of multiple linear regression (MLR) model was constructed as follows:

(1) pEC50=−5.987+1.987×ES_Sum_sssCH−0.812×ES_Count_aaN+8.608×BIC−2.047×IAC_Mean+0.812×CHI_3_P+2.159×JY.

**Figure 1:**
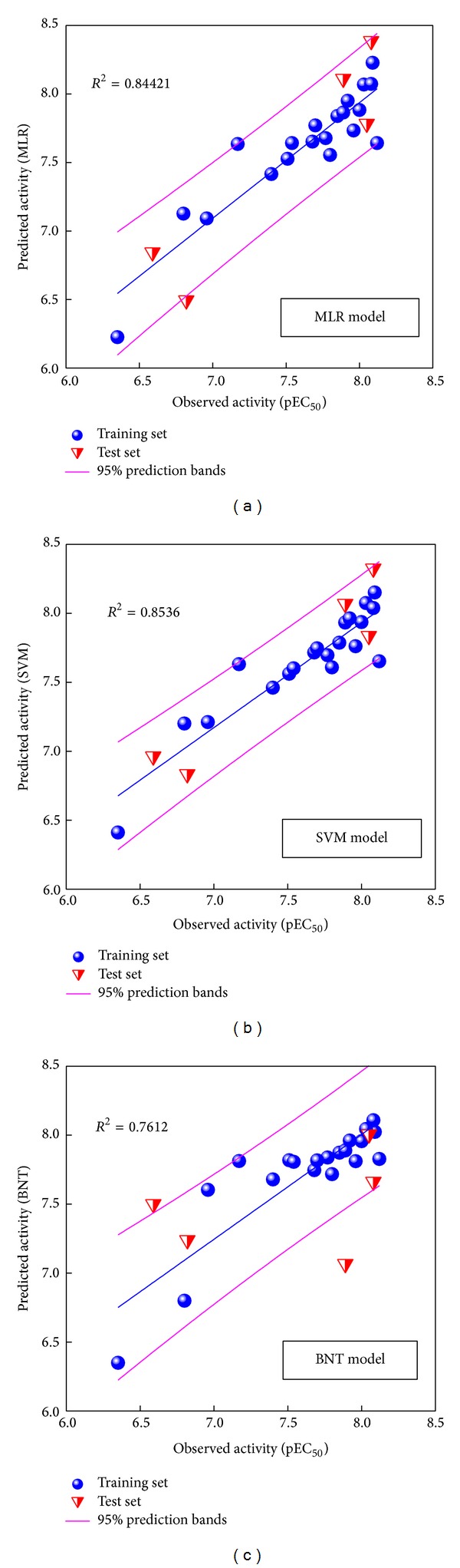
Comparative plots of observed versus predicted activity for (a) MLR, (b) SVM, and (c) BNT models. Correlation trend (blue line) and 95% prediction boundaries (enclosed by magenta lines) were shown.

The support vector machine (SVM) and Bayes network toolbox (BNT) models were also constructed with the identical training set and descriptors. The correlation of predicted and observed activities shown in Figure 1 illustrates the correlation trend and 95% prediction bands for each prediction model. The square correlation coefficients (*R* ^2^) of training set for MLR, SVM, and BNT models are 0.8442, 0.8536, and 0.7612, respectively. These prediction models are acceptable for predicting activity of PPAR-*γ* protein.

### 3.2. Docking Simulation

**Figure 2:**
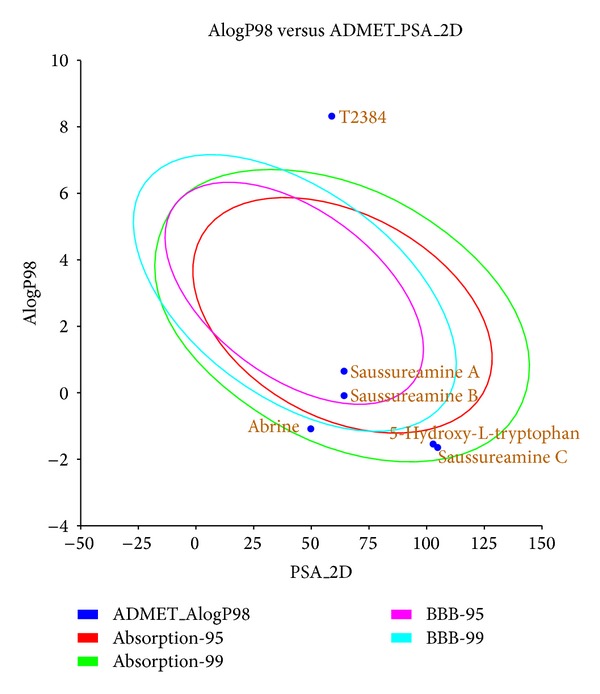
Human intestinal absorption model for top TCM compounds and T2384.

The potent compounds, which have acceptable predicted activities in all three prediction models, have been virtual screening with the target protein. After filtering by the absorption properties, the top TCM candidates ranked by Dock score were listed in Table 1 with their predicted activities and pharmacokinetics properties. Human intestinal absorption model displayed in Figure 2 suggested that the top five TCM candidates may have good absorption.

**Figure 3:**
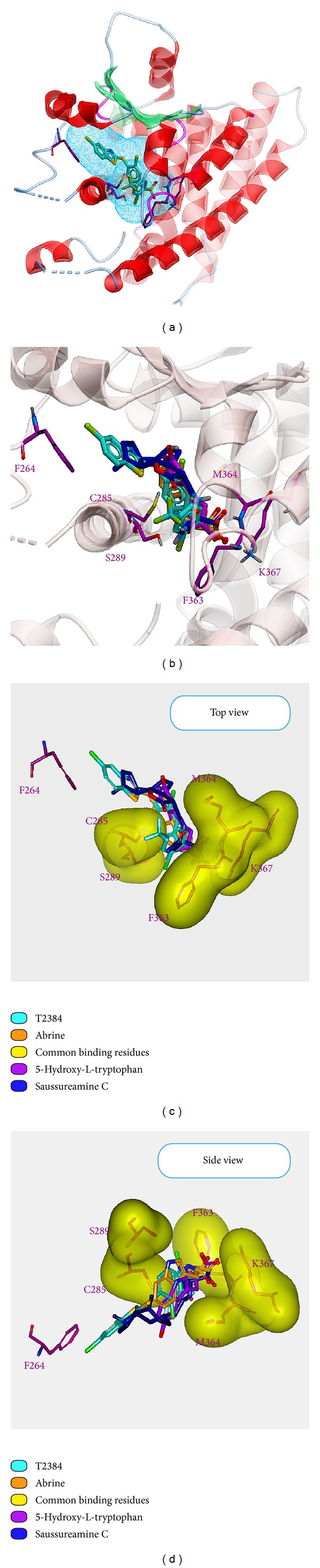
Binding sites and common binding residues for PPAR-*γ* protein. PPAR-*γ* protein with (a) binding site defined by T2384, (b) docking poses of top TCM compounds and T2384 in the binding site. (c) Top view, (d) side view of docking poses with common binding residues.

**Figure 4:**
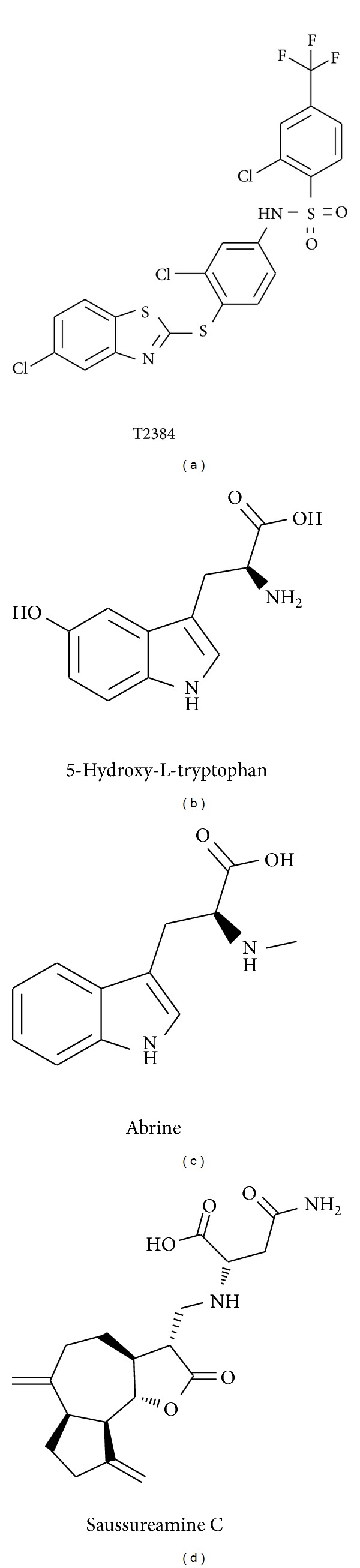
Chemical scaffold of control and top three candidates: (a) T2384, (b) 5-hydroxy-L-tryptophan, (c) abrine, and (d) saussureamine C.

**Figure 5:**
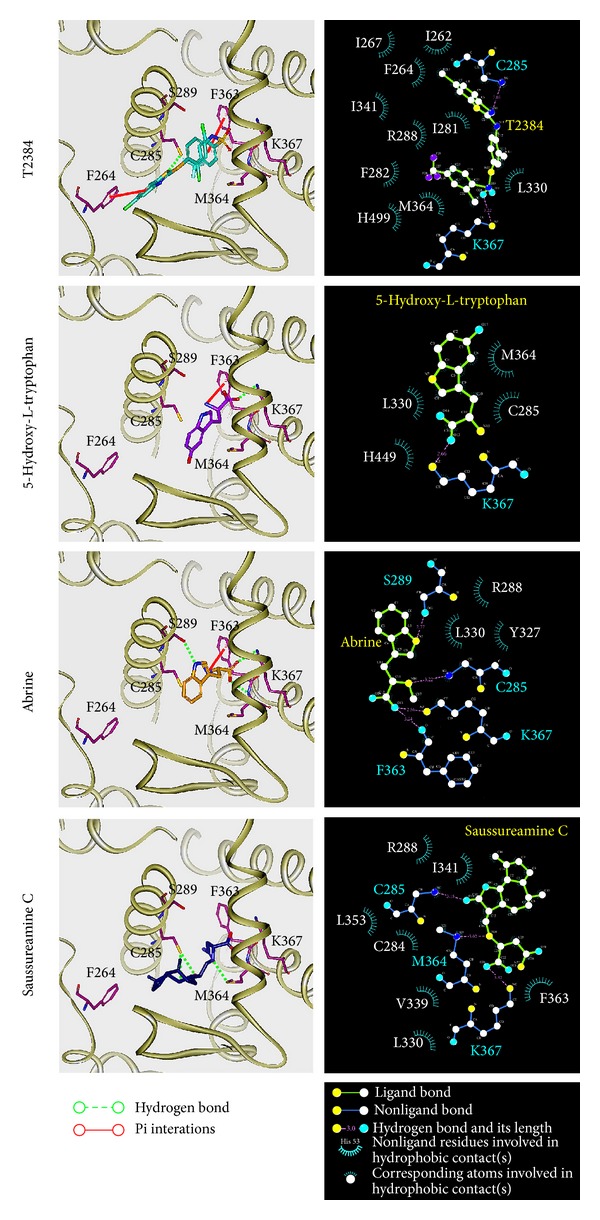
Docking pose of PPAR-*γ* complexes with T2384, 5-hydroxy-L-tryptophan, abrine, and saussureamine C, respectively. Hydrophobic contacts between PPAR-*γ* protein and each compound determined by LIGPLOT program.

For the docking simulation, the binding site of PPAR-*γ* protein was defined by the volume and position of control, T2384 (Figure 3(a)). We visually inspected docking poses of top ranked TCM candidates (Figure 3(b)), 5-hydroxy-L-tryptophan, abrine, and saussureamine C interacting with similar PPAR-*γ* binding site residues as control (Figures 3(c)-3(d)).  Figure 4 displays the structure of T2384 and top three candidates. According to the docking poses shown in Figure 5, T2384 has *π* interactions with residues Phe264 and Phe363, hydrogen bonds (H-bonds) with residues Cys285 and Lys367, and hydrophobic contact with other nine residues.

Compared with T2384 in PPAR-*γ* protein, the top three TCM candidates have been docked with similar docking poses. Due to the molecular size of three TCM compounds, none of them have interaction with Phe264 as T2384. Except saussureamine C, both of 5-hydroxy-L-tryptophan and abrine have *π* interaction with residue Phe363 as control. However, saussureamine C still has hydrophobic contact with residue Phe363. All top three candidates have similar H-bond with residue Lys367 and hydrophobic contacts with some common residues, such as Leu330 and Met364. Except that 5-hydroxy-L-tryptophan has hydrophobic contact instead of H-bond with residue Cys285, the other two candidates have the similar H-bond with residues Cys285 as T2384. In addition, abrine and saussureamine C also have H-bond with Ser289 and Met364, respectively.

### 3.3. Molecular Dynamics Simulation

The docking poses in the docking simulation illustrate that the top three TCM candidates have similar interactions with the target proteins as T2384. However, the structure of PPAR-*γ* protein is fixed during the progress of docking simulation. As this reason, the molecular dynamics (MD) simulations for each protein-ligand complex were performed to investigate the stability of interactions between ligand and target protein in the docking pose under dynamic conditions and investigate the possible variations for each protein-ligand complex after docking.

**Figure 6:**
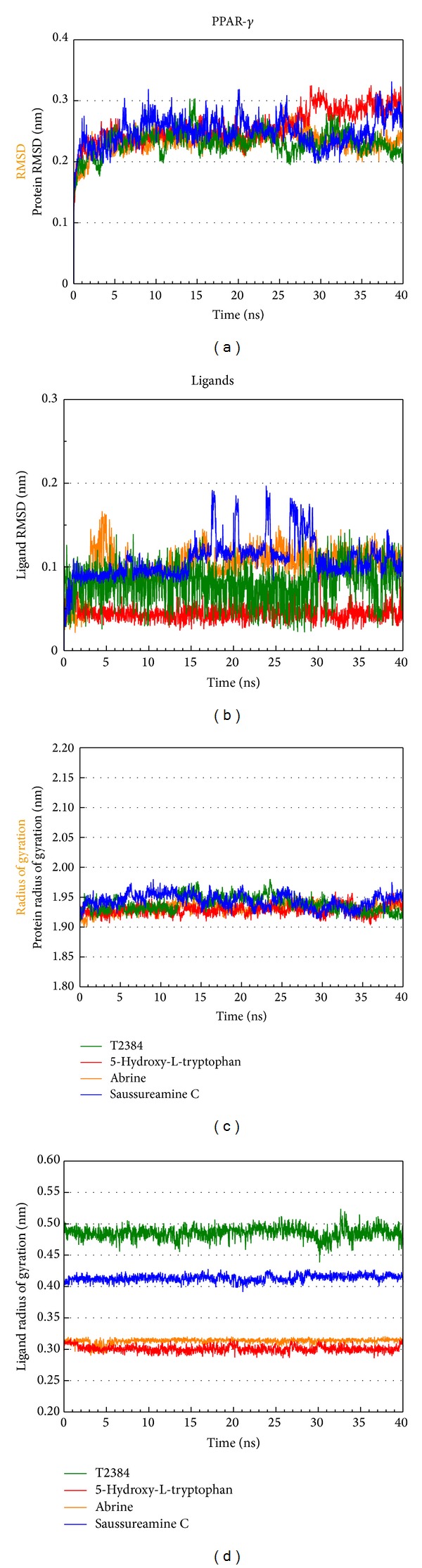
RMSDs and radii of gyration for PPAR-*γ* protein and ligands over 40 ns MD simulation.

**Figure 7:**
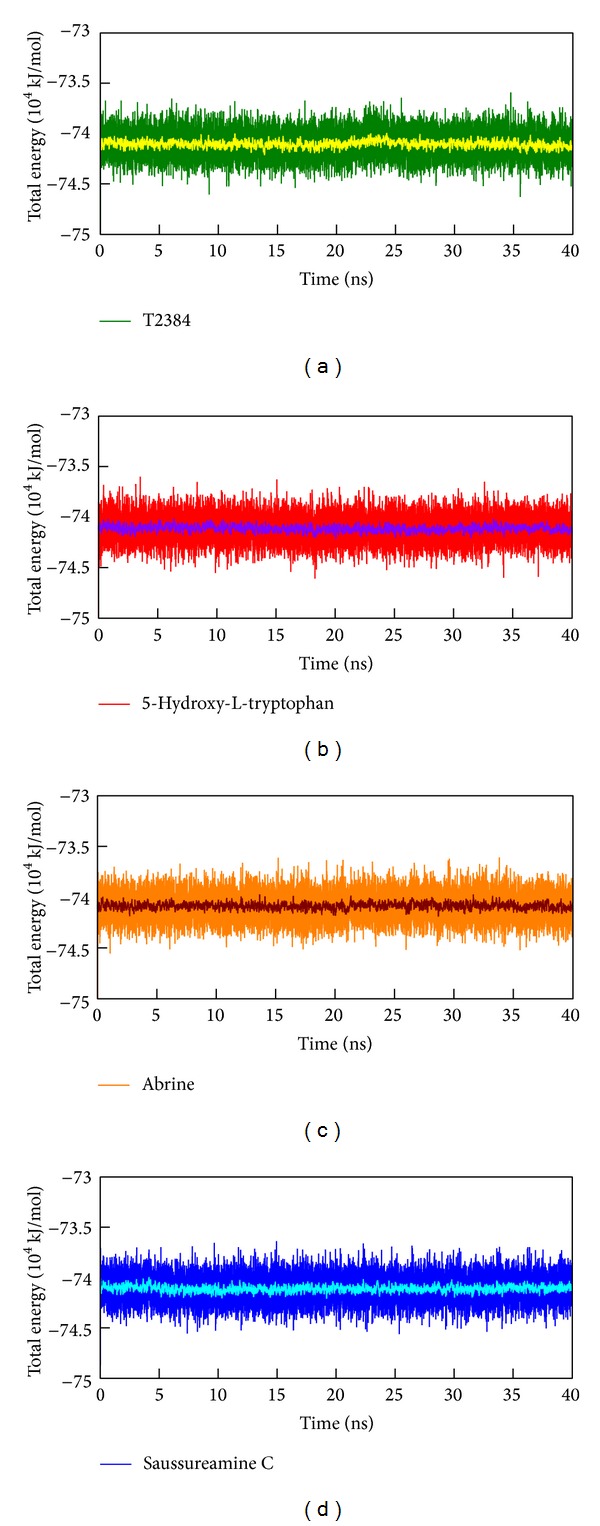
Total energy of PPAR-*γ* complexes with (a) T2384, (b) 5-hydroxy-L-tryptophan, (c) abrine, and (d) saussureamine C over 40 ns MD simulation. The average fluctuations in a cycle of 21 frames were illustrated by yellow (T2384), violet (5-hydroxy-L-tryptophan), wine (abrine), and cyan (saussureamine C) line, respectively.

**Figure 8:**
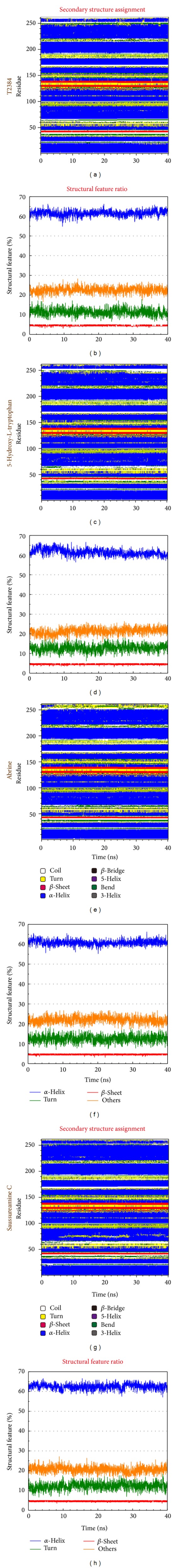
Secondary structure assignments and secondary structural feature ratio variations of PPAR-*γ* complexes over 40 ns MD simulation. Residues 1–65 in *y*-axis correspond to residues 207–271, residues 66-250 in *y*-axis correspond to residues 276–460, and residues 251–262 in *y*-axis correspond to residues 465–476.

The root mean square deviations (RMSDs) and radii of gyration for each protein and ligand in the complexes were illustrated in Figure 6. For RMSD, it calculates the deviation of the structure compared with the starting structure over 40 ns of MD simulation. They indicate that all protein-ligand complexes tend to be stable after 30 ns of MD simulation. Radius of gyration, which measures the mass of the atom relative to the center of mass of the complex, is indicative of the compactness of each complex. As shown in Figure 6, there is no significant variation for the compactness of each complex.  Figure 7 illustrates the variation of total energy for each protein-ligand complex over the course of 40 ns MD simulation with the average fluctuations in a cycle of 21 frames shown in the center of each graph. Total energy trajectories indicate that these systems were stabilized for PPAR-*γ* protein in the complex with T2384 and top three TCM candidates over the course of 40 ns MD simulation.  Figure 8 displays the variation of secondary structure of PPAR-*γ* protein and secondary structural feature ratio over the course of 40 ns MD simulation for each complex with T2384 and top three TCM candidates. It indicates that docking with three TCM candidates may not cause the significant differences from docking with the control in the secondary structure of PPAR-*γ* protein.

**Figure 9:**
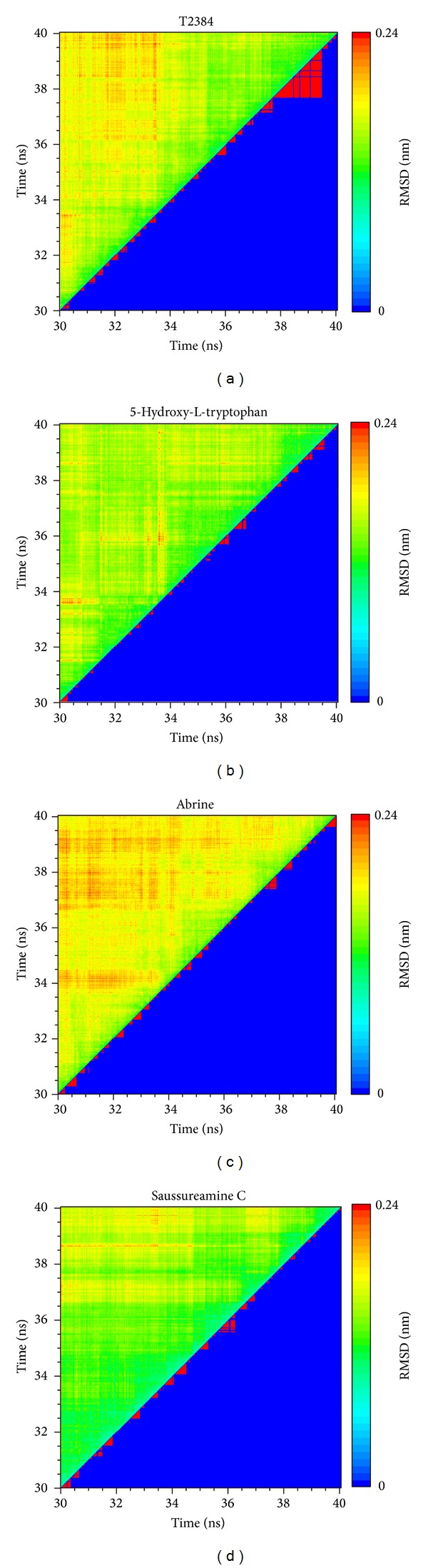
RMSD values (upper left half) and graphical depiction of the clusters (lower right half) of PPAR-*γ* complexes during 30–40 ns MD simulation.

**Figure 10:**
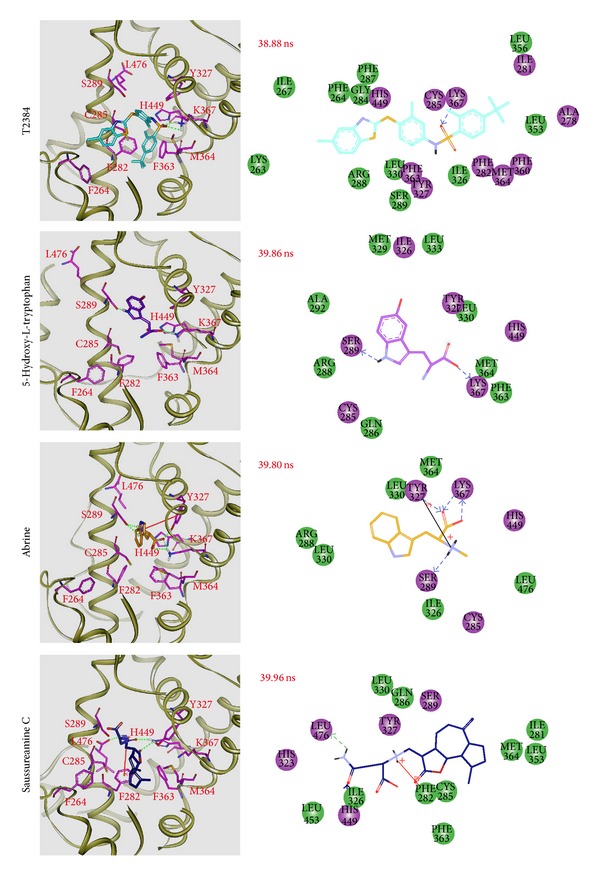
Docking poses of middle RMSD structure in the major cluster during 30–40 ns of MD simulation. Snapshots and ligand interaction diagrams for PPAR-*γ* protein complexes with T2384 (38.88 ns), 5-hydroxy-L-tryptophan (39.86 ns), abrine (39.80 ns), and saussureamine C (39.96 ns). For 2D diagrams, residues with magenta cycles are involved in hydrogen-bond, charge, or polar interactions, and residues with green cycles are involved in van der Waals interactions.

The representative structures of each complex after MD simulation were identified by the cluster analysis with a RMSD cutoff of 0.1 nm. In Figure 9, it illustrates the RMSD values and graphical depiction of the clusters over 30–40 ns MD simulation. The representative structures of each complex were identified by middle RMSD structure in the major cluster over 30–40 ns MD simulation, which are 38.88 ns (T2384), 39.86 ns (5-hydroxy-L-tryptophan), 39.80 ns (abrine), and 39.96 ns (saussureamine C), respectively. The snapshots and ligand interaction diagrams for each docking pose of the representative structures are illustrated in Figure 10. For T2384, it maintains the H-bonds with residues Cys285 and Lys367 in a nonstatic condition, which may retain the docking pose of T2384 in the binding pocket of PPAR-*γ* protein. In addition, the ligand interaction diagram also indicates that T2384 has interactions with common residues in docking simulation. For 5-hydroxy-L-tryptophan, it keeps the H-bond with residue Lys367 in a nonstatic condition and also has an H-bond with residue Ser289 as the docking pose of abrine in the docking simulation. Similarly, abrine has H-bonds with residues Ser289 and Lys367 as well as has an H-bond and *π* interaction with residue Tyr327. The docking pose of saussureamine C in the docking simulation is not stable in a nonstatic condition. In the representative structures after MD simulation, it has H-bonds with residues His449 and Leu476, as well as a *π* interaction with residue Phe282.

**Figure 11:**
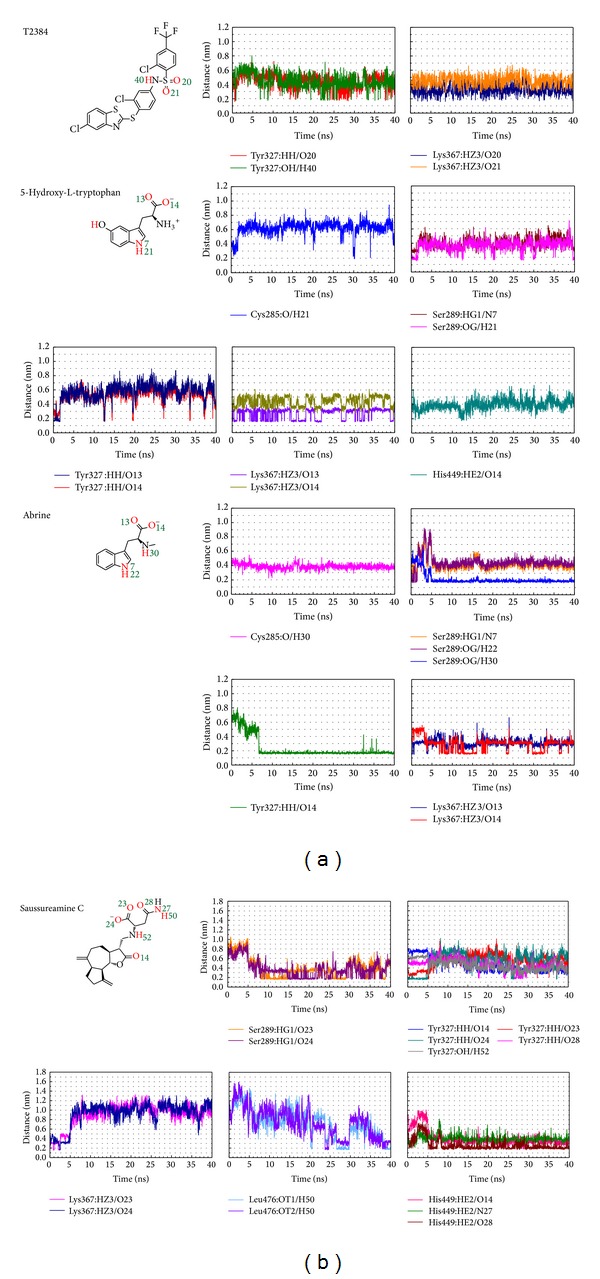
Distances of potential H-bonds between PPAR-*γ* protein and each compound during 40 ns MD simulation.

The H-bonds occupancies for key residues of PPAR-*γ* protein in each complex are shown in Table 2 with cutoff of 0.3 nm.  Figure 11 displays the variation of these distances over the course of 40 ns MD simulation. For T2384, the potential H-bonds with key residues of PPAR-*γ* protein are formed by its sulfonamide group. 5-Hydroxy-L-tryptophan and abrine form H-bonds with residue Lys367 by the carboxyl group. They form H-bonds with residue Ser289 by the indole group in the beginning of MD simulation, but the H-bond for abrine has shifted from the indole group to secondary amine group after 5 ns of MD simulation. In addition, the carboxyl group of abrine also forms a stable H-bond with residue Tyr327 after 7 ns of MD simulation. For saussureamine C, the docking pose in the docking simulation had changed after MD simulation. The H-bonds formed by the carboxyl group are shifted from residue Lys367 to residue Ser289 after 5 ns of MD simulation. In addition, it forms stable H-bonds with residue His449 by its sulfonamide group and heterocycle group after MD simulation.

**Figure 12:**
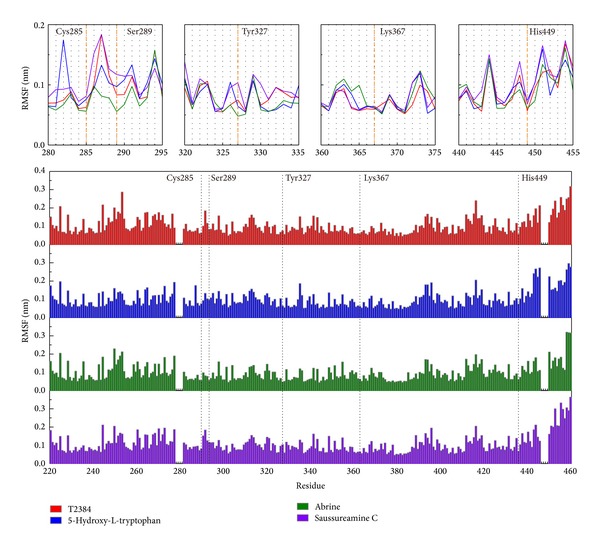
RMSFs for residues 207–476 of PPAR-*γ* complexes with each compound over 30–40 ns MD simulation. Common binding residues were illustrated with dash lines.

The root mean square fluctuations (RMSFs) shown in Figure 12 illustrate the stability of each residue over 30–40 ns MD simulation. Residues Cys285, Lys367, and His449 are stabilized by all top three TCM candidates and T2384. As abrine forms stable H-bond with residues Ser289 and Tyr327, the RMSFs of Ser289 and Tyr327 are much lower in the complex with abrine than with others. For saussureamine C, as the H-bonds with residue Tyr327 are shifted between the heterocycle group, secondary amine group, and sulfonamide group, it causes the highest value of RMSF for residue Tyr327 in the complex with saussureamine C.

**Figure 13:**
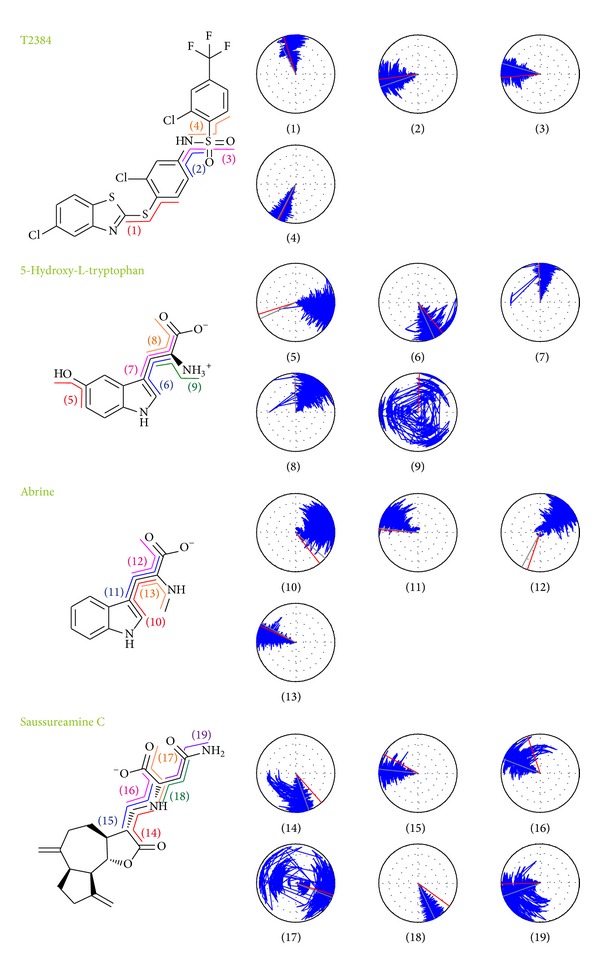
Variation of ligand torsion angles for each of PPAR-*γ* complexes during 40 ns of MD simulation. Red and gray lines represent the ligand torsion angle at docking simulation and first conformation of MD simulation, respectively.

To consider the variation of each ligand during MD simulation, variation of torsion angles during 40 ns of MD simulation for each ligand in the PPAR-*γ* complexes is shown in Figure 13. As T2384 is the cocrystallized compound in the PPAR-*γ* protein, the docking pose is stable during 40 ns of MD simulation. For 5-hydroxy-L-tryptophan, the docking pose which is also stable during 40 ns of MD simulation except for the hydroxyl group in the indole ring has a 180-degree shift after MD simulation. For abrine, the variation of torsions 10 and 11 at the initial period of MD simulation may be the reason that the H-bond has shifted from the indole group to secondary amine group, and carboxyl group forms a stable H-bond with residue Tyr327 after MD simulation. Torsions 14 and 16 for saussureamine C indicate that the docking pose of saussureamine C has a fluctuation during 15–30 ns of MD; it can also be seen in the ligand RMSD (Figure 6) and the distance variation with residue Tyr327 (Figure 11). The variation of torsion 19 shows that the sulfonamide group of saussureamine C is flexible over MD simulation.

## 4. Conclusion

This study aims to investigate the potent TCM candidates for PPAR-*γ* protein. The biologically activities of candidates were predicted by three distinct prediction models (MLR, SVM and BNT) based on their ligand characteristics. After docking simulation, the docking poses of top TCM compounds ranked by the scoring function were validated by the MD simulation. For the top three TCM candidates, both of 5-hydroxy-L-tryptophan and abrine have an indole ring and carboxyl group to form the H-bonds with the key residues of PPAR-*γ* protein. The secondary amine group of abrine also stabilized an H-bond with residue Ser289. The key residues were stabilized in protein complexes with 5-Hydroxy-L-tryptophan and abrine as control. For saussureamine C, the interactions of docking pose in the docking simulation are not stable after MD simulation. Hence, we propose 5-hydroxy-L-tryptophan and abrine as potential lead compounds for further study in drug development process with the PPAR-*γ* protein.

## Figures and Tables

**Table 1:** Docking results, predicted pEC_50_, and ADMET properties for top TCM compounds and T2384.

Name	Dock score	Predicted pEC_50_			CYP2D6^a^ probability	Hepatotoxicity probability	PPB level^b^
		MLR	SVM	BNT			
5-Hydroxy-L-tryptophan	148.721	5.89	6.62	6.59	0.069	0.291	0
Abrine	142.592	6.31	6.63	6.44	0.049	0.642	0
Saussureamine C	135.304	5.90	7.27	7.91	0.415	0.450	0
Saussureamine B	124.688	8.85	7.63	8.00	0.356	0.708	0
Saussureamine A	103.030	7.59	7.81	7.64	0.336	0.754	0
*T2384	**77.618**	**7.52**	**7.06**	**8.50**	**0.069**	**0.953**	**2**

*Control.

^a^Inhibition probability of cytochrome P450 2D6 enzyme.

^b^Plasma protein binding: 0: binding is <90%; 1: binding is >90%; 3: binding is >95%.

**Table 2:** H-bond occupancy for key residues of PPAR-*γ* protein with top three candidates and T2384 overall 40 ns molecular dynamics simulation.

Name	H-bond interaction	Occupancy
T2384	Tyr327:HH/O20	8.70%
	Tyr327:OH/H40	5.55%
	Lys367:HZ3/O20	39.30%
	Lys367:HZ3/O21	0.75%

5-Hydroxy-L-tryptophan	Cys285:O/H21	1.30%
	Ser289:HG1/N7	7.05%
	Ser289:OG/H26	0.15%
	Ser289:OG/H21	14.54%
	Tyr327:HH/O13	5.25%
	Tyr327:HH/O14	4.55%
	Lys367:HZ3/O13	49.93%
	Lys367:HZ3/O14	0.90%
	His449:HE2/O14	7.60%

Abrine	Cys285:O/H30	1.00%
	Ser289:HG1/N7	1.45%
	Ser289:OG/H22	3.00%
	Ser289:OG/H30	90.05%
	Tyr327:HH/O14	83.15%
	Lys367:HZ3/O13	41.15%
	Lys367:HZ3/O14	37.75%

Saussureamine C	Ser289:HG1/O23	32.40%
	Ser289:HG1/O24	30.05%
	Tyr327:HH/O14	4.95%
	Tyr327:HH/O23	6.20%
	Tyr327:HH/O24	14.65%
	Tyr327:HH/O28	6.60%
	Tyr327:OH/H52	6.65%
	Lys367:HZ3/O23	2.65%
	Lys367:HZ3/O24	2.70%
	His449:HE2/O14	14.65%
	His449:HE2/O24	3.50%
	His449:HE2/N27	3.90%
	His449:HE2/O28	77.60%
	Leu476:OT1/H50	13.85%
	Leu476:OT2/H50	7.00%

H-bond occupancy cutoff: 0.3 nm.
